# Handheld laser speckle contrast imaging probe: quantitative video rate assessment of tissue perfusion in animal and patient applications

**DOI:** 10.1117/1.BIOS.2.3.035003

**Published:** 2025-09-27

**Authors:** Han Dong, Parker A. Willmon, Pratheepa K. Rasiah, Sean T. Fitzgerald, Emmanuel A. Mannoh, Carmen C. Solórzano, Anita Mahadevan-Jansen

**Affiliations:** a Vanderbilt University, Vanderbilt Biophotonics Center, Nashville, Tennessee, United States; b Vanderbilt University, Department of Biomedical Engineering, Nashville, Tennessee, United States; c Vanderbilt University Medical Center, Department of Surgery, Nashville, Tennessee, United States

**Keywords:** laser speckle contrast imaging, intraoperative, perfusion, handheld, miniaturized, motion artifact, quantitative

## Abstract

**Significance:**

Laser speckle contrast imaging (LSCI) is a quantitative and contrast-free imaging technique for the perfusion status of superficial tissue blood vessels. This technique has been effectively used for perfusion assessment in many clinical applications. However, existing LSCI systems are typically overhead camera-based devices that are bulky and pose challenges for seamless integration into clinical workflow.

**Aim:**

Here, we present a miniaturized handheld LSCI system that has been validated on a flow phantom. Further, its intraoperative performance on tissue perfusion detection is compared with that of a conventional overhead LSCI system.

**Approach:**

The LSCI probe has a pen-shaped sterilizable package that allows for maneuverability. The performance of the handheld device was tested in mouse craniotomy and human parathyroidectomy experiments.

**Results:**

The developed handheld LSCI probe can differentiate perfused from compromised, unperfused tissue targets with performance similar to that of an overhead camera system.

**Conclusions:**

Miniaturized LSCI can classify the perfusion in both animal cortices and human parathyroid glands without the need for significant motion correction, suggesting easy adoption in future clinical applications.

Statement of Translation This work presents a handheld laser speckle contrast imaging probe that quantitatively assesses tissue perfusion during surgeries. Its usage has been proven in mouse surgery as well as determining the parathyroid glands’ viability during thyroid-related surgery. This tool informs the surgeon whether tissues/organs in the patient’s body are still viable before causing post-surgical complications.

## 1 Introduction

Laser speckle contrast imaging (LSCI) is an objective and label-free technique used to evaluate the perfusion of superficial blood vessels in tissues.^1^^,^^2^ It utilizes the speckle pattern generated by the interference of coherent light of a scattering medium. This interference varies randomly in space and produces a characteristic intensity pattern. The movement of scatterers within the medium causes fluctuations in constructive and destructive interferences, which causes a time-dependent variance of the speckle pattern measured by a photodetector. When these fluctuations are faster than the exposure time of the photodetector, blurring of the speckle pattern occurs. The level of blurriness directly relates to the relative speed of the particles moving within the medium and can be characterized by the correlation time as well as speckle contrast. Local blurriness of the speckle pattern image is quantified with the spatial contrast, $K_{s}$, defined as the standard deviation of the local pixel intensities, $\sigma_{s}$, divided by their mean intensity ⟨I⟩

$$K_{s}=\frac{\sigma_{s}}{\left\langle I\right\rangle }.$$ (1)

A spatial contrast map is therefore generated by convolving Eq. (1) to the raw speckle image with a specified sampling window^1^ and displays the presence of flow in the image. This produces a map of flow in the field of view. Speckle contrast is linked to the correlation time (τ_c), which depends on sample motion and camera exposure time. In theory, τ_c can be used to estimate flow velocity. In practice, this is difficult because the number of moving particles, their scattering properties, and their orientation are unknown.^1^^,^^3^ Recent studies show that traditional speckle contrast imaging measures the product of flow speed and vessel diameter, not speed or volumetric flux alone. With correction, relative red blood cell (RBC) speeds from speckle data differ by only 10±3% *in vivo* from RBC tracking.^2^ Given these considerations, this study employed the contrast value (K) as a qualitative indicator of vasculature, while recognizing that it lacks a direct correspondence to absolute flow speed.

LSCI is widely used across various medical applications to monitor blood circulation in various organs such as the retina,^4^^–^^6^ skin,^7^^–^^10^ brain,^11^^–^^14^ joints,^15^ and visceral organs.^16^ In previous work, our lab has demonstrated the utility of LSCI for detecting parathyroid gland viability with high sensitivity and specificity in the classification of perfused and compromised parathyroid glands during thyroid surgery.^17^ However, the camera system used in the study is a bulky overhead system, and the participating surgeons reported a preference for handheld probe-based devices that can access hard-to-reach areas, reducing the need for larger incisions. A compact and lightweight handheld guidance system would benefit surgeons by providing point-of-care decision support and readily accessible reference information.^18^^–^^20^ Thus, there exists a need for a miniaturized LSCI system that can assess the perfusion of the parathyroid glands during surgery, especially in small incisions.

Several reports have been published on the development of compact LSCI systems by utilizing miniaturized cameras,^21^ imaging fibers,^22^^–^^24^ and a chip-on-tip camera^25^ to reduce the system’s size, as the camera component is often the critical factor in determining overall dimensions. The miniaturized on-chip camera can effectively conduct wide-field LSCI to assess blood perfusion, both in a flow model and in the context of alterations in finger blood flow. However, the reported system was limited to extremity imaging and not for handheld use.

In designing a handheld LSCI device, motion has been shown to be a challenge in effective implementation and needs to be addressed. Motion artifacts in a handheld LSCI device have been shown to decrease the dynamic range of speckle contrasts throughout the field of view and thus reduce differentiability between perfused and unperfused pixel values.^26^^,^^27^ Efforts have been made to correct motion artifacts in handheld laser speckle images by utilizing an array of techniques. Lertsakdadet et al.^28^ show the feasibility of using a fiducial marker (FM), as an indicator of the level of movement artifact of the frame, to realize handheld LSCI. Chizari et al.^27^^,^^29^ studied the influence of camera orientation on different movement artifacts, such as tilting and translation in handheld LSCI. Their team also performed handheld LSCI on psoriasis lesions and showed comparable findings of handheld LSCI with FMs and proper post-frame registration.^30^ Farraro et al.^31^ developed a tablet LSCI handheld system that allows for point-of-care perfusion measurement. However, this approach required a set of 250 images for one acquisition over a period of 5 s as an acceptable tradeoff in the implementation of the handheld untethered LSCI device. Rege et al.^32^ prototyped an LSCI device that exhibited similar performance between stationery and handheld scenarios in assessing retinal blood flow. The LSCI prototype was pressed against the target tissue, and the physical contact reduces the motion that would otherwise have existed. These methods reduce motion artifacts in LSCI through post-processing or by minimizing motion during image capture, both of which prove effective.

The goal of this work is to develop a motion-resistant handheld system that produces video rate LSCIs. The system’s ability to detect perfusion *in vivo* was evaluated against a previously reported stationary overhead LSCI system.^33^ Handheld system performance was validated by comparing speckle contrast from each system of live and dead mouse cortices as well as human parathyroid glands before and after ligation. The results indicate that our handheld LSCI system is effective in detecting perfusion in small tissues, thereby providing a reliable miniaturized tool for evaluating the presence of microcirculation in both animal research and clinical settings.

## 2 Material and Methods

### 2.1 LSCI Systems

This study compares the performance of a standing overhead LSCI system, previously reported by Mannoh et al.,^33^ to the newly developed handheld probe system. The overhead system uses a 785-nm diode laser (Innovative Photonics Solutions, Monmouth Junction, New Jersey, United States), coupled through a single-mode fiber optic patch cord (Thorlabs, Newton, New Jersey, United States) that couples light from the laser to a lens tube attached to the exterior of the imaging head. This lens diverges the laser light to an ∼30  mm diameter spot at 400 mm from the lens. The maximum irradiance at the tissue surface was measured to be $∼6.8\;\mathrm{mW}/{\mathrm{cm}}^{2}$. An imaging lens system (Optotune, Dietikon, Switzerland) is used to focus the image onto the sensor of a near-infrared optimized camera (acA1300-60 gmNIR, Basler AG, Ahrensburg, Germany) with a 5-ms exposure time for the raw speckle acquisition.

**Fig. 1:**
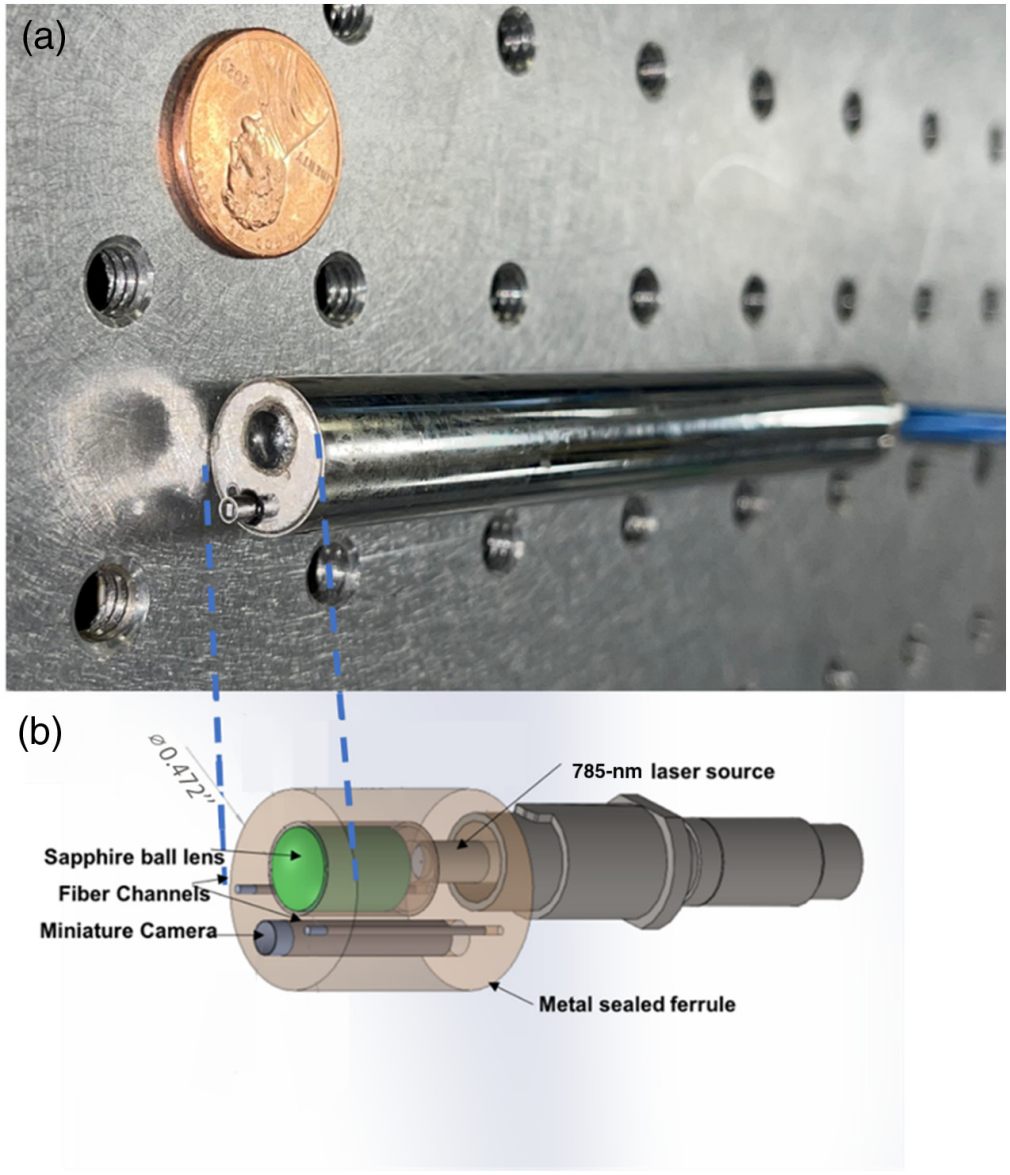
Clinical handheld LSCI probe-based system. (a) Picture of the probe. (b) Schematic of the imaging end.

A miniaturized handheld probe system was designed and manufactured as shown in Fig. 1. The probe uses the same 785-nm coherent light source (Innovative Photonics Solutions), coupled through a 400-μm polarization-maintaining fiber optic patch cord (Thorlabs) to a 6-mm focal length sapphire ball lens (Thorlabs). The ball lens diffuses light onto the surgical field of interest at ∼1  cm from the lens. The maximum laser irradiance at this distance was $2.5\;\mathrm{mW}/{\mathrm{cm}}^{2}$. A commercial miniature camera (Naneye2D F/2.7, ams OSRAM AG, Premstätten, Austria) was used to record the speckle pattern produced with a 5-ms exposure time for the raw speckle acquisition. The miniature camera field of view was measured to be 2  cm×2  cm when the probe tip was 1 cm above the target. A polarizer was placed in front of the camera, set perpendicular to the illumination polarization to reduce specular reflections. White light and perfused speckle contrast (k) image recorded by both systems were processed and displayed on the same computer.

### 2.2 Fluidic Phantom Design

An optical tissue phantom was employed to assess the efficacy of the newly designed LSCI system in generating valuable data while incorporating hand motion. This phantom was constructed using polydimethylsiloxane infused with titanium dioxide to replicate tissue scattering characteristics (reduced scattering coefficient of $8\;{\mathrm{cm}}^{-1}$ at 785 nm). A solution containing 0.4 g of milk powder diluted in 50 mL of water would pass through a 400-μm channel in the phantom. Subsequently, at least 30 image frames were captured across three trials using the overhead setup, stationary probe, and handheld probe under the following flow conditions: no flow, 1  mm/s, and 100  mm/s—mimicking a range of blood flow speeds from the capillaries to the arteries.^34^^,^^35^

### 2.3 Animal Study

In animal studies, three C57BL/6 mice (n=3) were anesthetized with isoflurane (induced with 3% isoflurane and maintained with 1.5% throughout the experiment) and stably positioned in a stereotaxic frame (Harvard Apparatus, Holliston, Massachusetts, United States), approved by Institutional Animal Care and Use Committee (IACUC, M2100052-00). A heating pad was used to maintain a body temperature of 36°C to 37°C throughout the experiments. The exposed region was continually bathed with sterile saline to maintain hydration.

Motion artifacts occur due to factors such as breathing, heartbeat, and the hand movements of the operator holding the probe. To avoid excessive movement on the speckle images, operators were instructed to maintain the field of view by placing the overhead standing system and holding the probe steady for an extended period during acquisition. At least 30 frames were taken for both the overhead system and the handheld probe of the cortex after the surgical procedure (perfused) and 5 min after euthanasia (compromised). The room lights were on during the image acquisition, whereas the surgical headlight and overhead light were turned off.

A motion threshold algorithm is applied to both *in vivo* studies to get rid of frames with excessive motion. The code first takes the average of the frame’s contrast value, which correlates to the blurring of the frames. This value can characterize the motion artifact included in the specific frame. Using the average contrast value as a threshold (0.025), we deselect frames with an average contrast value lower than this threshold, which correlates to excessive motion artifact.

### 2.4 Human Study

Five patients (n=5) undergoing parathyroidectomy at the Vanderbilt University Medical Center were recruited, and written informed consent was obtained before participation following Institutional Review Board (181543) approval. As part of the standard procedure, the surgeon ligated the blood supply to the diseased parathyroid gland before excision. The same imaging procedure was conducted for parathyroid perfusion assessment as in the animal study to confirm device operation in human subjects. Speckle contrast images were acquired from the parathyroid gland before ligation and then after the surgeon ligated the blood supply to the gland in preparation for removal. In addition, as part of the standard care protocol, the surgeon took intraoperative parathyroid hormone measurements before and after excision to confirm the removal of the hyperactive parathyroid. Imaging parameters for the perfused and ligated states and the motion threshold algorithm were acquired in a similar fashion to that of the animal study.

### 2.5 Speckle Contrast Calculation

Speckle contrast was calculated using Eq. (1), convolving the image of the acquired speckle pattern. A 5×5  pixel window is scanned across the acquired speckle pattern at each pixel location, and the speckle contrast is calculated as the standard deviation of pixel intensity values within the window divided by the average. The resultant speckle contrast value thus forms the new contrast (K) imaging. Values closer to 0 indicate regions of higher vascular flow, whereas higher values represent regions of lower vascular flow. In general, speckle value is inversely related to the flow speed of the fluid, such as blood flow within perfused tissues.^1^ The measured speckle contrast is taken to be a measurement of the sum of blood flow within microvessels on the surface of the tissue. Images are processed by MATLAB R2020a (The MathWorks Inc., Natick, Massachusetts, United States).

### 2.6 Analysis

For all studies, the first 30 non-selective frames of each trial were analyzed. For each frame, the region of interest (ROI) is first determined based on the white light image, and the speckle contrast is averaged over the area of interest spatially. Subsequently, the ROI’s spatially averaged speckle contrast over 30 frames was compiled into groups. For the phantom study, the data were grouped by the overhead system, the stationary probe, and the handheld probe with various flow speeds (0, 1, and 100  mm/s). For the animal study, the data were categorized into perfused and compromised, which were before and after the animal had been sacrificed. For the human study, the data were categorized into perfused and compromised, which were before and after the ligation of the parathyroid gland. Note that the motion rejection algorithm is only applied to human study.

As this study is focused on detecting the presence of flow, statistical analysis used a two-sample, two-sided Student’s t-test to compare ROI’s speckle contrast between perfused and compromised (flow and no flow) with GraphPad Prism v9.4.0 (GraphPad Software, Inc., La Jolla, California, United States).

## 3 Results

Due to its easy maneuverability and smaller form factor, the handheld probe-based system demonstrates superior compatibility with the surgical workflow compared with an overhead system. For comparison purposes, the overhead device previously reported by Mannoh et al.^33^ was employed alongside the handheld LSCI probe. Both systems are capable of video rating LSCI acquisition of the field of interest and differentiating perfused and unperfused targets in all studies while rejecting less than 5% of the frames with the motion threshold algorithm.

### 3.1 Phantom Study for Hand Motion Artifact Evaluation

**Fig. 2:**
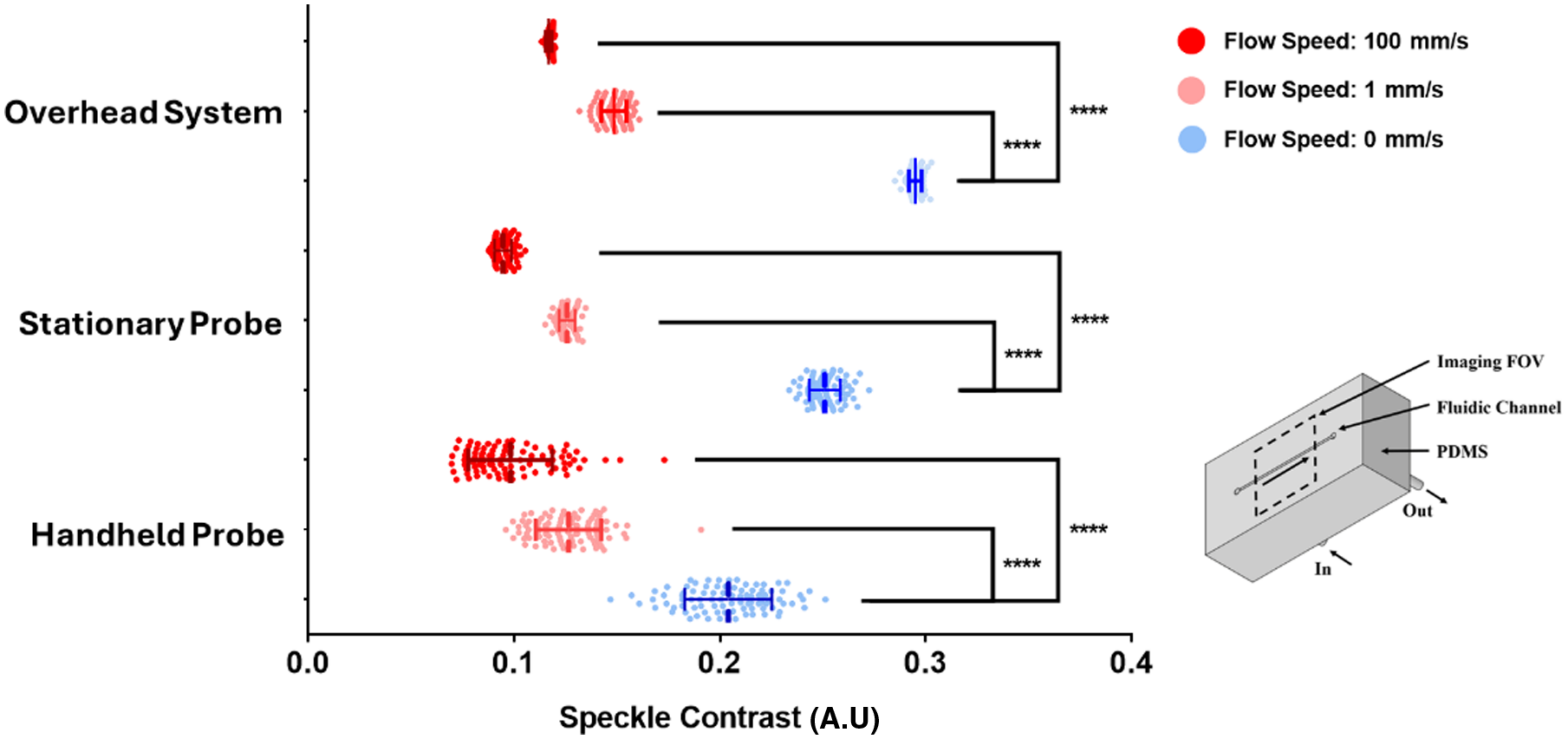
Speckle contrast of the tissue phantom channel for the overhead system, stationary, and handheld probe systems with various flow speeds (n=3) with 5-ms exposure time. t-tests were performed on scenarios where there is flow versus no flow (flow speed = 0 mm/s), and all groups resulted in high significance. Note: speckle contrast is inversely related to the flow speed/volume; thus, the lower speckle contrast represents faster flowing.

The phantom shown in Fig. 2 was used to evaluate the performance of three conditions: overhead camera system, stationary probe, and handheld probe with 5-ms exposure time. Imaging on the perfused fluidic channel, the handheld probe results in a contrast average with a standard deviation of 0.127±0.0157 for the 1  mm/s and 0.0979±0.0203 for the 100  mm/s, which distinguishes itself from a contrast average of 0.204±0.0210 of the channels with no flow. Stationary probe imaging on perfused phantom results in a contrast average of 0.126±0.00388 for the 1  mm/s and 0.0948±0.00401 for the 100  mm/s and a contrast average of 0.251±0.00743 with no flow. The overhead system on perfused phantom results in a contrast average of 0.149±0.00456 for the 1  mm/s and 0.117±0.00138 for the 100  mm/s and a contrast average of 0.295±0.00306 with no flow.

### 3.2 *In Vivo* Perfusion Assessment of Overhead Versus Handheld LSCI System

#### 3.2.1 Animal study

**Fig. 3:**
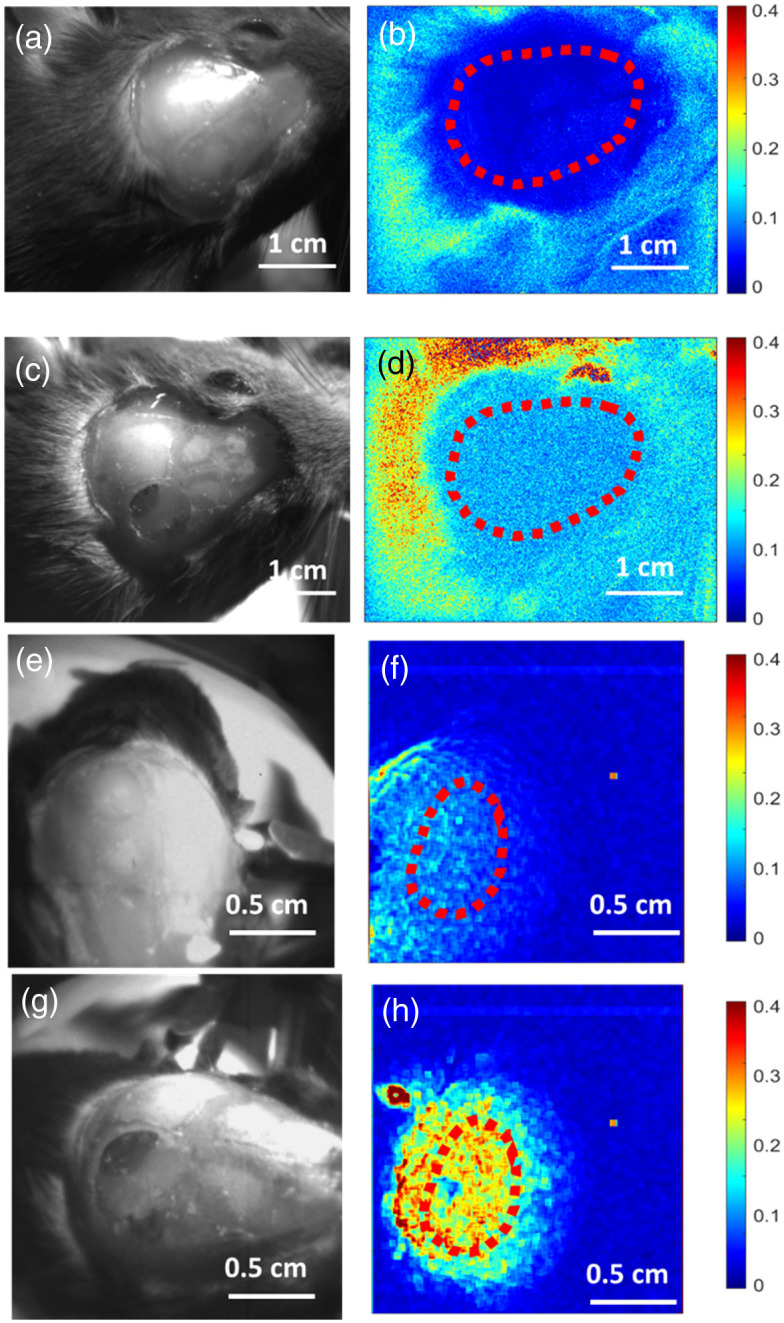
Representative white light and laser speckle contrast images of craniotomy of the somatosensory cortex of mice with an ROI drawn out with a red dotted line. (a) Overhead system’s perfused cortex white light images. (b) Overhead perfused speckle contrast image. (c) Overhead system’s compromised cortex white light image. (d) Overhead compromised speckle contrast image. (e) Handheld probe’s perfused cortex white light image. (f) Handheld perfused speckle contrast image. (g) Handheld probe’s compromised cortex white light image. (h) Handheld compromised speckle contrast image. Note: the color bar on the right indicates the value for the contrast images ranges from 0 (blue) to 0.4 (red), and the speckle contrast is inversely related to the flow speed.

Figure 3 presents the examples of perfused [Figs. 3(b) and 3(f)] and compromised [Figs. 3(d) and 3(h)] cortices imaged with the overhead system [Figs. 3(a)–3(d)] and handheld probe system [Figs. 3(e)–3(h)]. In this figure, the left column displays white light images of the craniotomy site, whereas the right column shows the corresponding speckle contrast image in false color using a color map ranging from 0 to 0.4. These images reveal that the perfused cortex exhibits visibly lower speckle contrast compared with the compromised cortex for both the overhead and handheld systems.

**Fig. 4:**
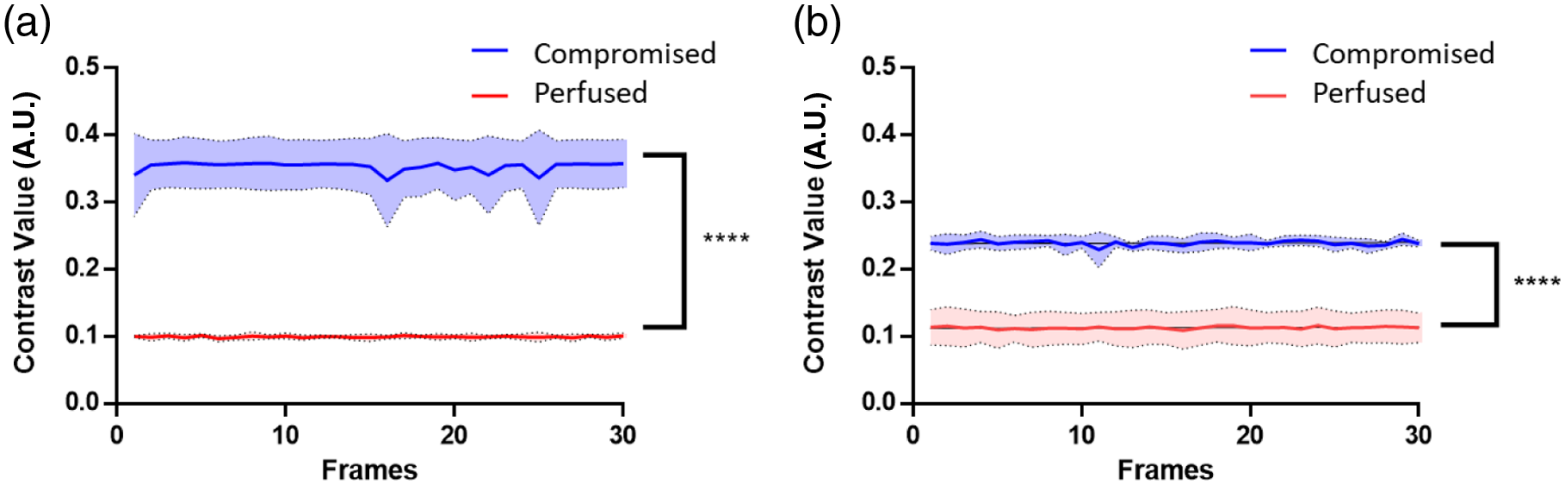
Average mouse cortex speckle contrast (0 to 0.5) grouped based on the vital status of the mice. (a) Overhead LSCI speckle contrast over 30 frames. (b) Handheld LSCI speckle contrast over 30 frames for perfused and compromised mouse cortex (in red versus blue, respectively). For each scenario, the average is presented as a solid line with a shaded area representing one standard deviation of over 30 frames. Speckle contrast is significantly lower (p<0.0001) for the perfused versus the compromised cortices in both overhead and handheld LSCI systems. Note: the graph is composed of the data from the first 30 frames of the video acquired at 40 frames per second.

The mean and standard deviation of spatial speckle contrast values of ROI (mouse cortex) in each frame (i.e., each time point) were calculated and plotted over 30 frames in Fig. 4 for both overhead and handheld systems. The average speckle contrast for perfused cortex with the overhead system was calculated to be 0.0945±0.0100 across all frames, compared with that of the compromised cortex at 0.354±0.0354 [Fig. 4(a)]. Similarly, the average speckle contrast for the perfused cortex with the handheld system was calculated to be 0.113±0.0208 compared with 0.239±0.00962 for the compromised cortex [Fig. 4(b)]. With both systems, the average speckle contrast was statistically significantly different (p<0.0001) between perfused and compromised tissues. Further, there was no overlap in the speckle contrast values of the two groups over multiple frames (i.e., time), demonstrating the ability of the handheld device to detect compromised versus perfused glands regardless of variability in the ROI contrast values that may be present due to potential motion.

#### 3.2.2 Human study

**Fig. 5:**
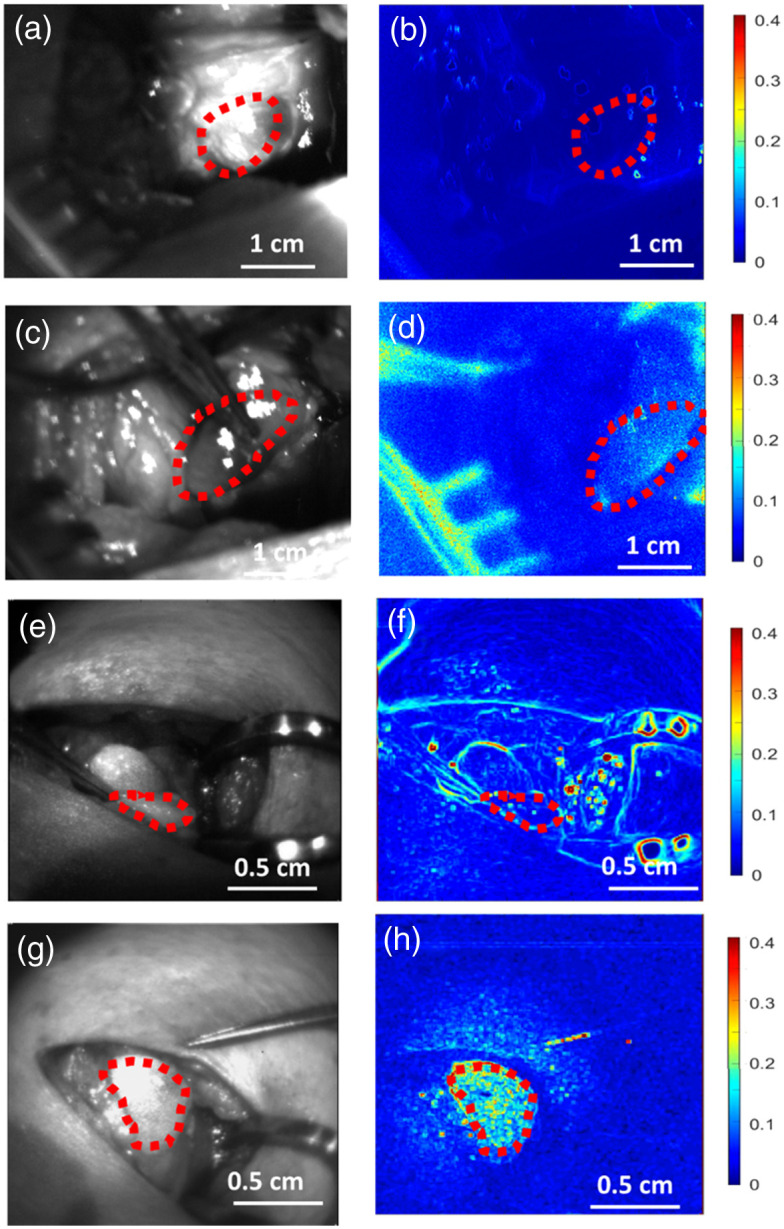
Representative white light (left column) and laser speckle images (right column) of the surgical field during parathyroidectomy (n=5). (a) and (b) White light and speckle contrast images of perfused parathyroid gland with overhead system. (c) and (d) White light and speckle contrast image of compromised parathyroid gland with overhead system. (e) and (f) Perfused parathyroid white light and speckle contrast images acquired with handheld probe. (g) and (h) Compromised parathyroid white light and speckle contrast images with a handheld probe. Note: the color bar on the right indicates the value for the contrast images ranges from 0 (blue) to 0.4 (red), and speckle contrast is inversely related to the flow speed.

Figure 5 shows the examples of parathyroid glands considered perfused and compromised based on visual assessment by the participating surgeons, obtained using both the overhead and handheld systems. The left column shows the white light images, whereas the corresponding speckle contrast images are in the right column. The parathyroid glands are outlined with red dashed contour lines.

**Fig. 6:**
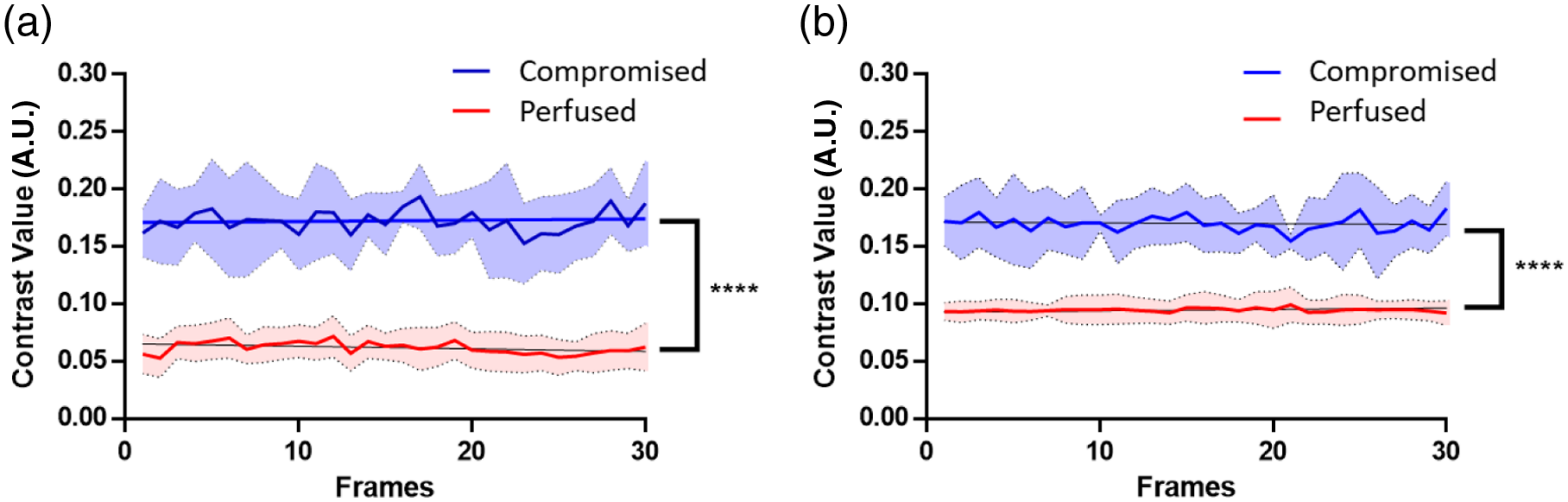
Average parathyroid speckle contrast values over frames, for perfused and compromised parathyroid glands as determined by a surgeon’s visual assessment. (a) Overhead LSCI speckle contrast over 30 frames (left) and (b) handheld LSCI speckle contrast over 30 frames (right) for perfused and compromised parathyroid glands (red versus blue, respectively) (n=5). The averages are presented as a solid line with the shaded areas representing the standard deviation over the 30 frames acquired. Speckle contrast is significantly lower (p<0.0001) for perfused parathyroid glands compared with compromised ones, for both overhead standing and handheld LSCI systems. Note: the graph is composed of the data from the first 30 frames of the video acquisition (40 frames per second).

The mean and standard deviation of spatial speckle contrast values of the ROIs were calculated using the first 30 frames for the five patients in Fig. 6. Across all 30 frames, the overhead imaging systems show a spatial contrast average of 0.0616±0.0153 for perfused parathyroid glands, which is distinct from the spatial contrast average of 0.173±0.0313 for compromised parathyroid glands. On the other hand, handheld imaging on perfused parathyroid glands results in a spatial contrast average of 0.09450±0.0100, which is distinct from the compromised parathyroid glands with a spatial contrast average of 0.170±0.0247. Statistically significant differences (p<0.0001) were observed among the glands categorized as perfused versus compromised using speckle contrast images acquired with both systems. Figure 6 demonstrates the ability of both systems to distinguish the perfusion status of the ROI on a frame-to-frame basis. This figure shows no overlap of the speckle contrast between the two groups over multiple frames (time) to demonstrate the ability of the handheld device to detect compromised versus perfused glands regardless of variability in the ROI contrast values due to motion.

## 4 Discussion and Conclusion

This work demonstrates the design of a miniaturized handheld LSCI probe and its ability to assess relative tissue perfusion in animal and human models. The performance of the handheld LSCI probe was validated in comparison with a stationary overhead camera-based LSCI system in an *ex vivo* setting using a flow phantom. Furthermore, the ability of the handheld LSCI probe to determine perfusion was evaluated in *in vivo* mouse cortex and human parathyroid glands. The results were validated with the ground truth provided by the overhead LSCI device, along with the opinion of the animal care personnel and the surgeon. The images obtained and the resultant analysis validate that the handheld LSCI probe developed is effective in differentiating perfused and compromised tissues with *in vivo* applications, offering a reliable new tool for intraoperative tissue vasculature assessment.

The handheld LSCI probe was designed as a pen-shaped device with an outer diameter of <1.2  cm and a length of 10 cm such that it can be comfortably held by a surgeon. A sapphire ball lens is placed in front of the illumination fiber to maximize overlap of the illumination and imaging field and create a spherical wavefront that is not as susceptible to rotational, tilt, and translation hand motion in comparison with other approaches, as demonstrated by Chizari et al.^29^ Such a design reduces motion artifacts generated by a probe that is handheld, allowing the biological motion, i.e., perfusion to be prominent in the handheld LSCI signal. When tested using a single-channel flow phantom, which mimics blood flowing through a vessel, the speckle contrast obtained from the handheld LSCI probe generates results comparable to that of the overhead LSCI system. Both LSCI systems demonstrate statistical significance in the presence and absence of flow in the channel, although the speckle contrast for the handheld probe is lower than that of the overhead system. It should be noted that different LSCI systems with distinct designs can have different speckle contrasts based on Eq. (2). Further, the speckle size for the LSCI system is calculated as

$$\rho_{\text{speckle}}=2.44\lambda (1+M)f/\#,$$ (2)

where λ is the wavelength of light, M is the magnification of the imaging system, and f/# is the f-number of the system. For the overhead and probe-based systems, the speckle size is ∼35 and 6  μm in diameter, respectively, whereas the pixel size for the camera used is 4.8  μm×4.8  μm, and 3  μm×3  μm, respectively. The difference in ratio between the speckle and detector sizes explains the difference in speckle contrast readout for the perfused and compromised targets. In addition, although both systems utilize cross-polarizers in their light source and camera, the extinction ratio of the polarizers is different, which also contributes to the difference in the raw speckle readout.

Despite the reduced speckle contrast, the results shown in Fig. 2 validate the ability of the handheld LSCI probe to withstand motion artifact and resolve flow in the single flow phantom. The results also show that the flow is comparable when the probe is mounted (and therefore stationary) to when the same probe is handheld. Although the handheld scenario exhibits a larger standard deviation in the speckle contrast compared with the stationary case, it yields a statistically significant difference between flowing and non-flowing phantoms. The larger variation in speckle contrast can be attributed to random hand motion, which introduces inconsistencies in the speckle pattern. Motion artifacts such as blurring and streaking introduce a global effect to the speckle contrast for the handheld LSCI probe in comparison with the stationary LSCI probe. As a result of this global motion, the contrast readout is expected to be lower when handheld compared with when the system is stationary, where such artifacts are absent, as observed in Fig. 2. In the perfused case, the speckle contrast shows the presence of blood flow regardless of handheld motion. Overall, LSCI with the handheld probe demonstrates its ability to differentiate flow versus no flow in the fluid channel of the phantom.

Intraoperatively, the handheld probe system shows a statistical significance in distinguishing the perfusion status of both mouse cortices and human parathyroid glands *in vivo*. The contrast images of the handheld LSCI probes are shown in Figs. 3 and 5 demonstrating that the probe can become a useful tool in checking tissue perfusion in clinical settings. Figures 4 and 6 also illustrate that the average speckle contrast of perfused tissues is significantly lower than that of the compromised tissues. These findings align with the concept that reducing blood flow corresponds to an increase in the speckle contrast in LSCI and the ability of the handheld system to distinguish between the two.

In comparison with other existing motion-resistant LSCI devices, the surgical pen-shaped system requires minimal post-processing due to its ergonomic design, reducing motion from handholding. The probe utilizes chip-on-tip technology that allows for 249×250 image resolution with a 1  mm×1  mm camera form factor. The small camera allows a small system form factor that can even act as an endoscope or laparoscope. The miniaturized camera and pen-shaped package allow the system to perform high-video-rate speckle contrast imaging without fiducial corrections, registration, or weighted contrast contribution analysis.^37^^–^^39^ A motion filtering is applied to the images acquired in the human study, where excessive motion frames were removed from the video stream. Out of the non-selective 30 frames of five patients, less than 5% of the frames were removed from the video stream (Fig. 6). In addition, the speckle contrast obtained with the handheld LSCI probe shows the same statistical significance (p<0.0001) for both perfused and compromised groups without the need for motion filtering.

Although the handheld LSCI probe is sufficient in assessing tissue perfusion, it is not spatially sensitive enough to distinguish blood vessels from the rest of the tissue, due to the design of the handheld probe. This could be attributed to the depth of imaging, which leads to a blurring of blood vessels that are buried within the tissue. In reflective LSCI Monte Carlo models, imaging quality rapidly decreases with imaging depth.^40^ There are reports on using methods such as transmissive-detected mode, employing dual-wavelength speckle illumination, or applying multiple exposure times to increase LSCI system sensitivity and, thus, blood vessel visibility.^39^^–^^42^ However, the need for detecting blood vessels or quantifying flow depends on the clinical need of the targeted application. Here, we present a solution to clinical problems where the need is limited to detecting the presence of perfusion. The probe-based system is also limited by uneven illumination. However, it has a minimum effect on speckle contrast across the field of view, as the contrast calculation intrinsically compensates for variations in intensity within the region of interest. These limitations will be addressed in future iterations of the handheld LSCI designs for better usage and integration into the clinic.

The benefits of this probe-based video rate LSCI device are particularly relevant for less experienced clinicians and residents in evaluating tissue perfusion levels. In the case of intraoperative assessment of the parathyroid in endocrine neck surgery, such a device can reduce hospitalization periods and long-term medication costs associated with post-surgical hypoparathyroidism.^36^^,^^43^^–^^45^ Moreover, in the event of small incision surgeries such as parathyroidectomies and thyroidectomies, the system offers a less disruptive alternative to the overhead system, facilitating a smoother surgical workflow. This device also holds potential applicability in other clinical procedures where there is a need to non-invasively investigate the presence of tissue blood perfusion, such as breast flaps, vessel grafts, and early-stage cancer detection.^46^^–^^48^ In addition, the probe system improves upon the overhead system with greater robustness and easy-to-use maneuverability in the clinic. The imaging procedure for the overhead system is more time-consuming compared with the imaging probe, on account of alignment and focusing. In addition, surgeons often must hold the articulating arm of the overhead system to make small modifications to the field of view and imaging distance. This demonstrates the necessity of an easy-to-use handheld LSCI probe as developed for this study.

## Data Availability

Processed data and processing code are available on GitHub: https://github.com/16hand/handheldLSCI (“16hand/handheldLSCI”). Raw data for the study can be shared upon request.
